# Dissecting tocopherols content in maize (*Zea mays* L.), using two segregating populations and high-density single nucleotide polymorphism markers

**DOI:** 10.1186/1471-2229-12-201

**Published:** 2012-11-02

**Authors:** Xu Shutu, Zhang Dalong, Cai Ye, Zhou Yi, Trushar Shah, Farhan Ali, Li Qing, Li Zhigang, Wang Weidong, Li Jiansheng, Yang Xiaohong, Yan Jianbing

**Affiliations:** 1 National Maize Improvement Center of China, China Agricultural University, Beijing, 100193, China; 2 Department of Bioinformatics, International Crops Research Institute for the Semi-Arid Tropics (ICRISAT), Hyderabad, India; 3 National Key Laboratory of Crop Genetic Improvement, Huazhong Agricultural University, Wuhan, Hubei 430070, China; 4 Cereal Crops Research Institute, Nowshera, Khyber Pukhtoonkhwa, Pakistan

**Keywords:** Maize, Tocopherols, QTL mapping

## Abstract

**Background:**

Tocopherols, which are vitamin E compounds, play an important role in maintaining human health. Compared with other staple foods, maize grains contain high level of tocopherols.

**Results:**

Two F_2_ populations (K22/CI7 and K22/Dan340, referred to as POP-1 and POP-2, respectively), which share a common parent (K22), were developed and genotyped using a GoldenGate assay containing 1,536 single nucleotide polymorphism (SNP) markers. An integrated genetic linkage map was constructed using 619 SNP markers, spanning a total of 1649.03 cM of the maize genome with an average interval of 2.67 cM. Seventeen quantitative trait loci (QTLs) for all the traits were detected in the first map and 13 in the second. In these two maps, QTLs for different traits were localized to the same genomic regions and some were co-located with candidate genes in the tocopherol biosynthesis pathway. Single QTL was responsible for 3.03% to 52.75% of the phenotypic variation and the QTLs in sum explained23.4% to 66.52% of the total phenotypic variation. A major QTL (*qc5-1/qd5-1*) affecting α-tocopherol (αT) was identified on chromosome 5 between the PZA03161.1 and PZA02068.1 in the POP-2. The QTL region was narrowed down from 18.7 Mb to 5.4 Mb by estimating the recombination using high-density markers of the QTL region. This allowed the identification of the candidate gene *VTE4* which encodes γ-tocopherol methyltransferase, an enzyme that transforms γ-tocopherol (γT)to αT.

**Conclusions:**

These results demonstrate that a few QTLs with major effects and several QTLs with medium to minor effects might contribute to the natural variation of tocopherols in maize grain. The high-density markers will help to fine map and identify the QTLs with major effects even in the preliminary segregating populations. Furthermore, this study provides a simple guide line for the breeders to improve traits that minimize the risk of malnutrition, especially in developing countries.

## Background

Vitamin E is the common name that describes eight naturally occurring compounds having tocopherol activity [1]. The eight compounds are lipid-soluble antioxidants with two distinct groups, tocopherols and tocotrienols. The two groups differ in the saturation of the side chain and vary in the number and location of methyl groups [2], and are classified according to the location of the methyl group: α-tocopherol (αT), β-tocopherol (βT), δ-tocopherol (δT), γ-tocopherol (γT), α-tocotrienol, β-tocotrienol, δ-tocotrienol and γ-tocotrienol [3,4]. Vitamin E plays an important role in plants development and can protect cell membranes from oxidation. Vitamin E can prevent oxidation of polyunsaturated fatty acid by absorbing the superfluous free radicals produced in the lipid peroxidation chain reaction [5,6]. This serves to remove the free radical intermediates, thereby preventing continuity of the oxidation reaction. Vitamin E can prevent several diseases in humans and other animals, such as cardiovascular disease, Alzheimer's disease, neurological disorders, cancer, cataracts, inflammatory diseases and age-related macular degeneration [1,7]. Food and nutrition guidelines recommend 15 mg/day of vitamin E for both adults and teenagers [8]. Individuals in developed nations can easily fulfill their daily requirement of vitamin E, but vitamin E deficiency (VED) in the developing countries is more common in premature infants and elderly people [9]. Furthermore, VED that is not immediately treated can lead to other serious diseases such as muscle weakness, ataxia, blindness, dementia, and eventually spinocerebellar degeneration [9-12].

**Figure 1:**
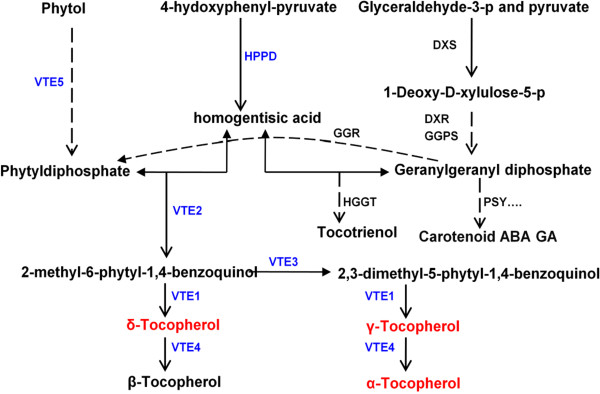
**Vitamin E synthesis pathway.** The three tocopherol compounds measured in this study are in red. Key enzymes in the pathway are labeled in bold type and the blue bold type indicates enzymes used for co-localization analysis. HPPD: 4-hydoxyphenyl-pyruvate dioxygenase; DXS: 1-deoxy-D-xylulose-5-phophate synthase; DXR: 1-deoxy-D-xylulose-5-phosphate reductase; GGPS: geranylgeranyl diphosphate synthase; VTE1: tocopherol cyclase; VTE2: HPT, homogentisic acid phytyltransferase; VTE3: 2-methyl-6-phytyl-1, 4-benzoquinol methyltransferase; VTE4: tocopherol methyltransferase; VTE5: phytol kinase; HGGT: homogentisate geranylgeranyl transferase; PSY: phytoene synthase.

The tocopherol biosynthesis pathway has been well studied in the model plant – *Arabidopsis* (Figure 1) [4]. Several enzymes that participate in the biosynthetic pathway have been characterized and annotated in *Arabidopsis* and *Synechocystis* PCC6803 including 4-hydoxyphenyl-pyruvate dioxygenase (HPPD/PDS) [13,14], tocopherol cyclase (VTE1/SXD1) [15,16], homogentisic acid phytyltrasferase (VTE2/HPT) [17-20], 4-benzoquinol methyltransferase (VTE3) [21,22], tocopherol methyltransferase (VTE4/γ-TMT) [23,24], and phytol kinase (VTE5) [25]. In *Arabidopsis*, the first step in the biosynthetic pathway is to form the – homogentisic acid (HGA) and phytyldiphosphate (PDP). HGA can be synthesized from 4-hydroxyphenyl-pyruvate by HPPD/PDS and PDP can be translated from phytol by VTE5. Through the function of geranylgeranyl reductase (GGR), PDP can also be synthesized from geranylgeranyl diphosphate (GGPP), which is also a precursor for carotenoid and tocotrienol biosynthesis. The second step in the pathway involves forming the immediate precursor, 2-methyl-6-phytyl-1,4-benzoqiunol (MBPQ) from HGA and PDP through VTE2/HPT. Third, VTE3 translates MBPQ into 2, 3-dimethyl-5-phytyl-1,4-benzoqiunol (DMBPQ), and then converts to γT. The last step involves the formation of different tocopherols using VTE1 and VTE4/γ-TMT enzymes. Many of the genes involves in the tocopherol synthesis pathways are similar in different plants and have been cloned in several plants, such as tomato and rice [26-28].

In addition to several other quality traits (such as oil and carotenoid), tocopherol content has been a focus of modern agriculture and several QTLs for tocopherol content has been mapped in different plant species [29-32]. Marwede et al. [29] used a double haploid (DH) population to locate several QTLs for γT, αT, TT and α/γ in winter oilseed rape. Elsa M and Vera-Ruiz et al. [30] performed fine mapping for the *Tph1* gene, which can lead to a sharp reduction in βT in sunflower F_2_ and F_3_ populations. To date, two maize QTL mapping studies of tocopherols have been conducted. Wong et al. [31] mapped several QTLs for tocopherols using an F_2:4_ segregating population (W64a × A632) and one test-cross population with AE335 using 123 simple sequence repeat (SSR) markers, and identified QTLs for γT, αT, TT and α/γ on chromosomes 1 and 5. Chander et al. [32] identified 31 QTLs in 16 regions covering all chromosomes except chromosome 4 by using a recombinant inbred line (RIL) population with 208 SSR markers. In these studies, the confident interval of identified QTLs was >10 cM because of the limited number of markers used.

Recently, a new genotyping technique, using the third generation marker system with SNP markers has been developed [33,34]. Compared with SSR markers, SNP markers are an ideal marker system because they are evenly distributed across the genome, are co-dominant, and accurate, and can be generated in a high-throughput and cost-effective manner. In this study, we used a maize GoldenGate assay containing 1536 SNPs to construct high-density linkage maps for two segregating populations with one common parent. The goals of this study were: (1) to identify QTLs affecting tocopherol content by analyzing two segregating populations with one common parent across the whole genome; (2) to fine map the major QTLs using the high-density markers; (3) and to explore the genetic architecture of tocopherol biosynthesis in different maize genetic backgrounds which could provide valuable information for further research.

## Results

### Phenotypic variation in tocopherols

Significant phenotypic variation was observed among the three parent lines in the traits of interest. For both γT and TT, the content was lowest in the common parent K22 (γT: 3.95 ± 0.15μg/g and TT: 9.18 ± 0.43μg/g), followed by CI7 (γT: 11.27 ± 0.68μg/g and TT: 18.09 ± 1.06μg/g) and was highest Dan340 (γT: 31.46 ± 0.42μg/g and TT: 34.51 ± 0.29μg/g). The αT content was highest in CI7 (6.76 ± 0.65μg/g), followed by the common parent K22 (5.13 ± 0.29μg/g), and was lowest in Dan340 (2.98 ± 0.21μg/g) which contains half the αT level of CI7. In contrast, α/γ ratio was highest in K22 (1.30 ± 0.03), followed by CI7 (0.60 ± 0.0) and was lowest in Dan340 (0.09 ± 0.01). The distribution of different tocopherols in the three parents also varied. In K22, the content of γT (3.95 ± 0.15μg/g)was just half as that of αT (5.13 ± 0.29μg/g) where as γT content in CI7 (11.27 ± 0.68μg/g) was nearly twice as that of αT (6.76 ± 0.65μg/g), even in Dan340 γT content (31.46 ± 0.42μg/g) was more than ten times as that of αT (2.98 ± 0.21μg/g) in Beijing location in 2009. And similar trends was also observed in Hainan location in 2009 (Table 1).

**Table 1:** **Means, range, and broad-sense heritability (*h***^***2***^**) for tocopherols related traits**

**Trait**	**K22**^**a**^	**CI7**^**a**^	**D**	**Populations**	**K22/CI7 population**			**K22/Dan340 population**		
	**Mean ± SE**	**Mean ± SE**	**Mean ± SE**		**Mean ± SE**	**range**	***h***^**2b**^	**Mean ± SE**	**Range**	***h***^**2b**^
γT	3.95 ± 0.15	11.27 ± 0.68	**31.46 ± 0.42**	09BJF_2:3_	11.02 ± 0.42	1.96-45.81	0.73	22.46 ± 0.76	2.70-66.28	0.81
	-	10.37	33.40	09HNF_2:4_	10.96 ± 0.56	0.71-43.27		22.32 ± 0.95	1.35-54.78	
	-	-	-	10HBF_2:4_	5.65 ± 0.22	1.42-17.95		12.12 ± 0.62	1.30-38.23	
αT	5.13 ± 0.29	**6.76 ± 0.65**	2.98 ± 0.21	09BJF_2:3_	8.24 ± 0.21	2.39-18.22	0.77	6.63 ± 0.22	0.67-23.73	0.59
	-	14.95	5.57	09HNF_2:4_	10.11 ± 0.27	1.88-19.93		6.66 ± 0.26	0.11-16.56	
	-	-	-	10HBF_2:4_	12.11 ± 0.3	3.71-25.14		8.08 ± 0.35	0.93-34.10	
TT	9.18 ± 0.43	18.09 ± 1.06	**34.51 ± 0.29**	09BJF_2:3_	19.24 ± 0.52	5.47-60.78	0.75	29.32 ± 0.78	7.30-74.58	0.79
	-	25.32	38.97	09HNF_2:4_	21.07 ± 0.68	4.11-60.43		29.16 ± 0.92	7.77-60.80	
	-	-	-	10HBF_2:4_	17.75 ± 0.44	5.26-33.87		20.97 ± 0.72	3.65-51.53	
α/γ	**1.30 ± 0.03**	0.60 ± 0.01	0.09 ± 0.01	09BJF_2:3_	0.94 ± 0.04	0.18-4.02	0.70	0.41 ± 0.02	0.02-2.70	0.68
	-	1.44	0.17	09HNF_2:4_	1.41 ± 0.08	0.07-5.83		0.63 ± 0.07	0.01-5.53	
	-	-	-	10HBF_2:4_	2.61 ± 0.08	0.19-6.30		1.09 ± 0.08	0.05-5.31	

^a^ Average of three repeats tocopherols contents of the three parents in 2009 Beijing; ^b^ Broad-sense heritability of the tocopherols in the two populations; *Note*: The unit for γT, αT, and TT is μg/g, the max contents for each trait were bold type.

For different traits, the level of phenotypic variation varied by several folds (αT in POP-1) to nearly hundred folds (α/γ in POP-2) changes. The mean for γT in POP-1(11.02 ± 0.42μg/g) was less than that in POP-2 (22.46 ± 0.76μg/g) and the range was also larger in POP-2.the similar phenomenon was observed in TT. However the mean value for αT and α/γ in POP-1 was higher than that in POP-2 and was also consistent with the observed value for the respective parents of each line (Table 1).

The broad sense heritability (*h*^*2*^) was estimated from the F_2:3_ data and the two corresponding F_2:4_ populations. The heritability for each trait was high but varied for different traits. Heritability was highest (0.81) for γT in POP-2 and lowest (0.59) was divulged for αT in POP-2 (Table 1). Pearson correlation coefficients for each trait in every generation showed that γT was significantly positively correlated (*r*=0.82-0.96) with the secondary trait-TT but showed little or even no correlated with αT (*r*= (−0.07)-0.47) (Additional file 1: Table S1). The correlation coefficients between the F_2:3_ populations and two F_2:4_ populations ranged from 0.45 to 0.57 in POP-1 and from 0.26 to 0.77 in POP-2 (Additional file 1: Table S2).

### Genetic linkage map

Among 1536 SNPs, 468 markers were polymorphic in POP-1 and 357 markers in POP-2. After deleting markers that were located in the same position or were unlinked, only 429 (POP-1) and 344 (POP-2) markers were used for linkage map construction (Table 2). Because of the common parent K22, the two maps had many common markers whereas, 619 unique markers were used to construct a consensus map following the method of Wu et al. (Additional file 1: Figure S1) [35]. The POP-1 map covered a 1389.3-cM region, smaller than that of POP-2 1567.5-cM region. The average interval distance between two markers was shorter in POP-1 (3.25 cM) than the POP-2 (4.57 cM). The integrated map covered a much larger region (1649.03 cM) than the two separate maps, with 619 SNP markers and a narrow interval distance of 2.67 cM (Table 2).

**Table 2:** Marker characteristics by chromosome for the two linkage maps and the consensus map

**Population**	**Description**	**chr1**	**chr2**	**chr3**	**chr4**	**chr5**	**chr6**	**chr7**	**chr8**	**chr9**	**chr10**	**EL**^**a**^
K22/CI7	Number of markers	55	39	48	45	55	45	37	44	36	25	429
	Length(cM)	202.3	132	154.1	144.9	194.4	138.6	105.5	135.7	104.6	77.2	1389.3
	Average interval(cM)	3.75	3.47	3.28	3.29	3.6	3.15	2.93	3.16	2.99	3.22	3.25
K22/Dan340	Number of markers	52	22	39	20	30	41	30	46	31	33	344
	Length (cM)	249.7	170.7	162.8	137.7	154.5	140.2	143.2	166.5	130.7	111.5	1567.5
	Average of interval	4.9	8.13	4.28	7.25	5.33	3.51	4.94	3.7	4.36	3.48	4.57
Common^b^	Number of markers	17	10	19	13	15	23	10	18	19	10	154
Consensus^c^	Number of markers	90	51	68	52	70	63	57	72	48	48	619
	Length(cM) (cM)	250.8	168	166.1	149	211.4	156.7	139.2	166.3	129.3	112.4	1649.03
	Average interval(cM)	2.82	3.36	2.48	2.92	3.06	2.53	2.49	2.34	2.75	2.39	2.67

^a^ entire linkage; ^b^ these markers exist in the two linkage maps; ^c^ the linkage map through integrating the two linkage maps into one map with the same markers.

### QTL mapping

After 1,000 permutation tests, the threshold logarithm of odds (LOD) scores were defined as 3.7 whereas 17 QTLs were detected in the POP-1 F_2:3_ and 13 QTLs in POP-2 F_2:3_ (Tables 3 and 4). In the Hainan environment, only seven QTLs were detected in POP-1 F_2:4_ and five in POP-2 F_2:4_. In Hubei, ten putative QTLs were observed for POP-1 and 12 for POP-2 (Tables 3 and 4). Seven QTLs for each of the POP-1 and POP-2 populations were detected in at least two environments. The major QTLs were confirmed in the three environments for the respective maps. In both maps the total QTLs detected were 30 and including 17 in POP-1 and 13 in POP-2. Most of the detected QTLs showed additive effects rather than dominate effects.

**Table 3:** QTLs for tocopherols in K22/CI7 population and related candidate genes

**Trait**	**Populations**	**Chr**	**QTL**^**a**^	**PK**^**b**^**(cM)**	Marker* interval	**Genetic interval (cM)**	**Physical interval**^**c**^**(Mb)**	**LOD**	**A**^**d**^	**D**^**e**^	**R**^**2f**^**(%)**	**Candidate gene**
γT	09BJF_2:3_	2	*qc2-1*	25.91	PZB00901.3-PZA03228.4	24.02-54.47	9.4-20.1	4.33	−1.84	−0.18	5.99	
		5	*qc5-2*	67.6	PZA01327.1-PHM16854.3	62.04-77.24	15.1-35.3	11.84	−3.9	0.58	17.61	
		5	*qc5-3*	183.2	PHM3612.19-PHM13639.13	196.03-207.23	213.3-215.8	3.98	1.91	−2.53	5.16	
		7	*qc7-1*	43.5	PZA03149.4-PZA02643.1	48.86-62.83	108.8-134.1	6.12	2.85	0.37	8.83	
	09HNF_2:4_	5	*qc5-4*	57.1	PZA01371.1-PZA01327.1	43.04-62.04	8.3-15.1	4.97	−2.81	−0.68	11.01	
		5	*qc5-5*	93.5	PZA00067.10-PZA00148.3	109.1-115.8	145.9-164.7	10.18	−2.56	−2.95	17.33	
	10HBF_2:4_	5	*qc5-2*	67.6	PZA01327.1-PHM16854.3	62.04-77.24	15.1-35.3	15.82	−2.66	0.67	25.02	
		5	*qc5-1*	159.7	PZA03161.1-PZA00545.26	144.6-177.68	186.4-207.7	5.53	1.12	0.31	7.75	*VTE4*
		6	*qc6-1*	6.81	PHM15961.13-PZA03069.8	7.0-25.9	9.5-83.0	3.82	−1.22	0.21	5.32	
αT	09BJF_2:3_	1	*qc1-1*	75.4	PHM2130.29-PHM1950.71	97.59-104.09	55.5-67.8	3.97	−1.05	0.79	3.88	
		5	*qc5-2*	59.1	PZA01327.1-PZB00869.4	62.04-74.57	15.1-33.1	5.3	−1.18	0.23	6.19	
		5	*qc5-1*	150.8	PZA00352.23-PZA02060.1	152.75-166.18	191.6-203.2	24	−2.23	−0.08	29.63	*VTE4*
		6	*qc6-2*	77.6	PZA02262.3-PZB01308.2	87.7-95.2	134.9-144.6	4.66	−0.79	−0.29	4.66	
	09HNF_2:4_	5	*qc5-2*	67.6	PZA03226.3-PZA02207.1	67.14-79.77	20.2-49.9	4.13	−1.57	0.11	6.11	
		5	*qc5-1*	150.8	PZA00352.23-PZA02060.1	152.75-166.18	191.6-203.2	12.26	−2.49	0.35	20.06	*VTE4*
	10HBF_2:4_	1	*qc1-1*	66.1	PZA00081.18-PZA03189.4	85.05-101.69	45.5-64.2	4.11	−1.04	−0.39	5.48	
		5	*qc5-2*	69.1	PZA01327.1-PHM16854.3	62.04-77.24	15.1-35.3	14.64	−2.73	−0.16	18.17	
		5	*qc5-1*	149.5	PZA00352.23-PZA02060.1	152.75-166.18	191.6-203.2	7.87	−1.85	−0.16	9.84	*VTE4*
TT	09BJF_2:3_	1	*qc1-1*	68.1	PZA00081.18-PHM1932.51	85.05-118.11	45.5-120	5.27	−3.2	1.85	7.07	
		2	*qc2-2*	20.91	PHM12952.13-PZB00901.4	12.9-25.32	4.9-9.4	4.09	−1.64	−1.05	5.99	
		5	*qc5-4*	54.1	PZA01371.1-PZA01327.1	43.04-62.04	8.3-15.1	6.49	−3.56	1.63	9.53	
		5	*qc5-2*	67.6	PZA02113.1-PHM13675.17	72.97-90.34	31.0-67.5	12.49	−4.6	0.48	16.87	
		5	*qc5-1*	150.8	PZA00352.23-PZA02060.1	152.75-166.18	191.6-203.2	4.34	−0.66	−2.21	5.37	*VTE4*
		7	*qc7-1*	41.6	PZA03149.4-PZA02643.1	48.86-62.83	108.8-134.1	5.31	2.61	1.11	6.9	
	09HNF_2:4_	5	*qc5-2*	67.6	PZA01327.1-PHM13675.17	62.04-90.34	15.1-67.5	14.89	−7.05	−0.89	26.51	
	10HBF_2:4_	5	*qc5-2*	63.9	PZA01327.1-PZA02207.1	62.04-79.77	15.1-49.9	11.24	−5.35	0.93	15.47	
α/γ	09BJF_2:3_	5	*qc5-2*	57.1	PZA01327.1-PHM2769.43	62.04-83.27	15.1-58.5	6.79	0.28	−0.08	9.17	
		6	*qc6-2*	59.2	PZA01729.1-PZA02328.5	60.5-76.8	123.7-137.1	4.85	−0.23	0.17	8.72	
		8	*qc8-1*	69.9	PHM934.19-PZA02011.1	69.68-82.39	118.2-141.6	4.17	−0.14	−0.06	5.51	
	09HNF_2:4_	5	*qc5-2*	66	PZA03298.1-PHM16854.3	68.87-77.24	21.9-35.3	5.75	0.54	0.12	10.67	
		5	*qc5-1*	145.5	PZA02751.1-PZA02513.1	151.05-165.68	190.7-203.3	9.08	−0.51	−0.28	18.37	*VTE4*
	10HBF_2:4_	5	*qc5-2*	67.6	PZA01327.1-PHM2769.43	62.04-83.27	15.1-58.5	10.52	0.74	−0.25	12.65	
		5	*qc5-1*	159.7	PZA00352.23-PZA02015.11	152.75-180.48	191.6-208.3	19.32	−0.81	−0.11	26.03	*VTE4*
		6	*qc6-2*	67.5	PZA00473.5-PZA02262.3	60.4-87.7	124.1-134.9	6.05	−0.56	0.3	8.29	

^a^ the QTL name which were defined only in our research; ^b^ the peak position with the highest LOD in the K22/CI7 map; ^c^ the physical distance from the website ( http://www.panzea.org/, B73_version 5a.60);^d^ additive effect of the corresponding QTL, A; ^e^ dominance effect of the corresponding QTL, D; ^f^ the ratio of phenotypic variance can be explained by the QTL; * the physical position of these markers can be obtained from the website ( http://www.panzea.org/, B73_version 5a.60);

Note: the effect of the alleles assumes that the favorable allele came from K22.

**Table 4:** QTLs for tocopherols in K22/Dan340 population and related candidate genes

**Trait**	**Populations**	**Chr**	**QTL**^**a**^	**PK**^**b**^**(cM)**	Marker* interval	**Genetic interval (cM)**	**Physical interval**^**c**^**(Mb)**	**LOD**	**A**^**d**^	**D**^**e**^	**R**^**2f**^**(%)**	**Candidate gene**
γT	09BJF_2:3_	1	*qd1-2*	131	PZA02750.3-PHM2187.34	119.28-127.7	102.6-157.1	4.52	−3.88	1.29	3.03	
		1	*qd1-1*	167	PZA02117.1-PHM4926.16	165.46-179.76	224.1-241.2	34.95	−11.16	3.86	30.81	
		5	*qd5-1*	98.5	PZA02751.1-PZA02068.1	151.05-169.78	190.7-205.3	15.05	−6.08	−0.15	12.06	*VTE4*
		8	*qd8-1*	81.6	PZA02748.3-PZA02011.1	68.92-82.39	118.7-141.6	6.5	4.44	−0.31	4.44	
	09HNF_2:4_	1	*qd1-1*	167	PHM3690.23-PZB01647.1	158.46-174.76	218.1-231.7	13.1	−10.53	2.7	23.5	
	10HBF_2:4_	1	*qd1-2*	49.8	PZA00887.1-PZA00358.12	38.74-49.57	11.0-19.0	5.31	−3.43	1.27	5.38	
		1	*qd1-1*	164	PHM3690.23-kip1.3	158.46-190.8	218.1-256.5	24.31	−6.25	0.47	32.63	
		2	*qd2-1*	67.7	PZA03228.4-PHM10404.8	54.47-70.52	20.1-40.5	3.79	−1.82	−0.5	4.06	
		5	*qd5-1*	98.5	PZA03161.1-PZA01265.1	144.6-163.15	186.5-202.0	14.35	−4.45	−1.39	18.2	*VTE4*
αT	09BJF_2:3_	5	*qd5-1*	98.5	PZA02751.1-PZA02068.1	151.05-169.78	190.7-205.3	21.88	3.58	−0.17	52.75	*VTE4*
		8	*qd8-1*	75.4	PZB00592.1-PHM4203.11	76.29-77.22	125.3-134.9	3.71	−1.36	0.59	5.01	
	09HNF_2:4_	5	*qd5-1*	96.5	PZA02751.1-PZA02068.1	151.05-169.78	190.7-205.3	5.63	1.89	0.39	12.44	*VTE4*
	10HBF_2:4_	1	*qd1-1*	169	PHM3690.23-kip1.3	158.46-190.8	218.1-256.5	7.3	−2.35	0.68	11.08	
		5	*qd5-1*	101	PZA03161.1-PZA02068.1	144.6-169.78	186.5-205.3	21.76	3.93	0.87	39.09	*VTE4*
		10	*qd10-1*	30.1	PZA01642.1-PZA00079.1	28.06-29.45	14.6-18.9	4.14	0.5	−1.96	5.37	
TT	09BJF_2:3_	1	*qd1-3*	106	PZA02292.1-PZA01267.3	89.29-109.03	51.3-77.2	4.9	−3.31	−0.36	3.97	
		1	*qd1-1*	167	PHM3690.23-PZB01647.1	158.46-174.76	218.1-231.7	42.07	−12.34	3.42	50.67	
		5	*qd5-2*	85.3	PZA01779.1-PZA00643.13	103.8-107.1	82.0-91.8	6.16	−2.22	−2.63	5.66	*HPPD-5*
		5	*qd5-1*	92.5	PZA03161.1-PZA01265.1	144.6-163.15	186.5-202.0	7.54	−3.29	−1.53	6.22	*VTE4*
	09HNF_2:4_	1	*qd1-1*	167	PHM3690.23-PZB01647.1	158.46-174.76	218.1-231.7	13.42	−10.49	2.9	25.28	
	10HBF_2:4_	1	*qd1-4*	49.8	PZA00887.1-PZA00358.12	38.74-49.57	11.0-19.0	4.72	−4.14	1.52	5.62	
		1	*qd1-1*	164	PHM3690.23-PZB01647.1	158.46-174.76	218.1-231.7	24.13	−8.54	1.2	42.48	
		2	*qd2-1*	67.7	PZA03228.4-PHM10404.8	54.47-70.52	20.1-40.5	3.74	−2.95	0.69	4.71	
α/γ	09BJF_2:3_	1	*qd1-1*	168	PHM3690.23-PHM4926.16	158.46-179.76	218.1-241.2	24.2	0.3	−0.15	26.67	
		5	*qd5-1*	101	PZA02751.1-PZA02068.1	151.05-169.78	190.7-205.3	24.26	0.33	−0.09	28.18	*VTE4*
		8	*qd8-1*	78.1	PZB00592.1-LYCE.1	76.29-79.82	125.3-138.8	4.28	−0.12	−0.02	4.32	
	09HNF_2:4_	1	*qd1-1*	167	PHM3690.23-PZB01647.1	158.46-174.76	218.1-231.7	9.8	0.68	−0.31	17.94	
		5	*qd5-1*	98.5	PZA02751.1-PZA02068.1	151.05-169.78	190.7-205.3	5.47	0.59	−0.31	10.56	*VTE4*
	10HBF_2:4_	1	*qd1-1*	166	PHM3690.23-PZB01647.1	158.46-174.76	218.1-231.7	3.61	0.46	−0.15	4.71	
		5	*qd5-1*	98.5	PZA03161.1-PZA02068.1	144.6-169.78	186.5-205.3	26.84	1.2	−0.22	51	*VTE4*

^a^ the QTL name which were defined only in our research in K22/Dan340; ^b^ the peak position with the highest LOD in the K22/Dan340 map; ^c^ the physical distance from the website ( http://www.panzea.org/, B73_version 5a.60);^d^ additive effect of the corresponding QTL, A; ^e^ dominance effect of the corresponding QTL, D; ^f^ the ratio of phenotypic variance can be explained by the QTL; * the physical position of these markers can be obtained from the website ( http://www.panzea.org/, B73_version 5a.60).

Note: the effect of the alleles assumes that the favorable allele came from K22.

**Figure 2:**
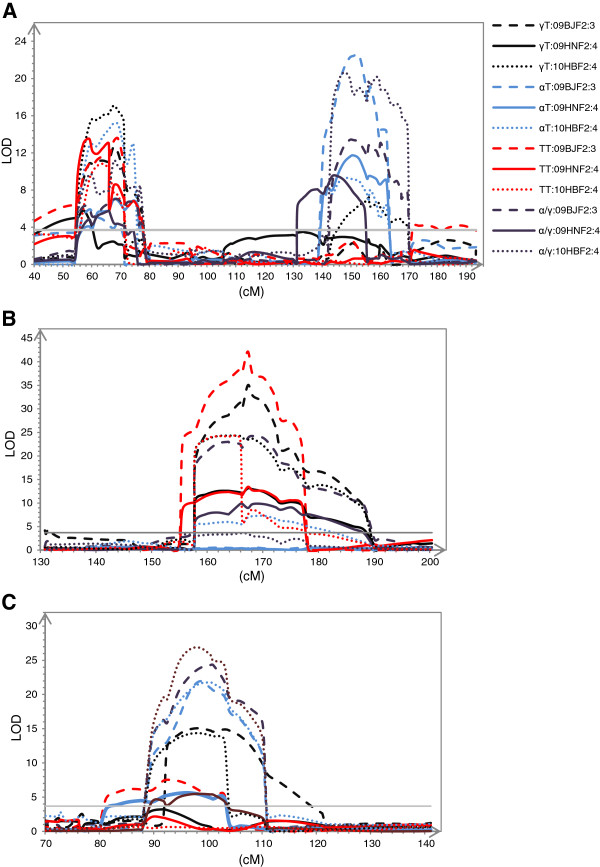
**Distribution of major QTLs in the two maps (A), The two major QTLs on chromosome 5 in the POP-1 map (40–190 cM genetic distance); (B) The major QTL on chromosome 1 in the POP-2 map (130–200 cM genetic distance); (C) The major QTL on chromosome 5 in the POP-2 map (70 to 140 cM genetic distance).** The symbol color of relates to the corresponding trait in each location beside the figure. Gray solid lines indicated the permutated threshold.

Among the 17 QTLs in the POP-1 F_2:3_, γT and αT had four each, whereas TT had six and α/γ had three (Table 3). These QTLs were mainly located in 11 regions on different chromosomes, with one each on chromosomes 1, 6, 7 and 8, two on chromosome 2 and four on chromosome 5. No QTLs were detected on chromosomes 3, 4, 9 and 10. Each QTL explained from 3.88% (αT on chromosome 1) to 29.63% (αT on chromosome 5) of the phenotypic variation. All the QTLs explained 37.59% (γT), 44.36% (αT), 51.73% (TT) and 23.40% (α/γ) of the total phenotypic variation for each trait. Two QTLs with a > 7 LOD were detected in the POP-1 map. The *qc5-1* was between the markers PZA03161.1 and PZA02068.1 (134.1-159.7 cM in the POP-1 map) affecting the content of γT, αT, TT and α/γ it explained 5.37% to 29.63% of the total phenotypic variation for each trait. The other QTL, defined as *qc5-2* were located between the markers PZA01327.1 and PHM1870.20 (55.1-76.7 cM in the POP-1 map), and affected the content of γT, αT, TT and α/γ; it explained 6.11% to 26.51% of the phenotypic variation for each trait (Figure 2A). The K22 allele of *qc5-1* on the long arm of chromosome 5 was responsible for decreasing the content of αT, TT and α/γ and for increasing γT. While to *qc5-2* at the short arm of chromosome 5, the favorable allele was from CI7 for γT, αT, TT, and K22 for α/γ (Table 3).

The 13 putative QTLs in POP-2 F_2:3_ were distributed as follows: four (γT), two (αT), four (TT), three (α/γ) (Table 4). These QTLs were located in five regions on different chromosomes; five QTLs each on chromosome 1 and 5, and three QTLs on chromosome 8. Three regions were on chromosome 1, and one each on chromosome 5 and 8. No QTL was observed on chromosomes 2, 3, 4, 6, 7, 9 and 10. Each QTL could explain the phenotypic variation from 3.03% (γT on chromosome 1; *qd1-2*) to 52.75% for αT on chromosome 5 (*qd5-1*, between PZA03161.1 and PZA02068.1, 144.60 – 169.78 cM; Table 4). All detected QTLs could explain 50.34% (γT), 57.76% (αT), 66.52% (TT) and 59.17% (α/γ) of the total variation for each trait. In POP-2 the *qd1-1* on chromosome 1 between PHM3690.23 and PHM4926.16 (158.5-179.76 cM in the POP-2 map) could explain 50.67% variation for TT, –whereas *qd5-1* could explain 52.75% phenotypic variation for αT (Table 4, Figure 2B and C). The Dan340 alleles at *qd1-1* were associated with increasing γT, αT and TT contents but did not affect α/γ, whereas the *qd5-1* alleles from K22 on the short arm of chromosome 5, had increasing (αT and α/γ) or decreasing (γT and TT) effects.

### Fine mapping of *qd5-1* in the POP-2 map

To dissect the large *qc5-1/qd5-1* QTL detected in both maps, the polymorphic markers at the threshold LOD near the peak were selected. The QTL contributed 29.63% (09BJF_2:3_), 20.06% (09HNF_2:4_), and 9.84% (10HBF_2:4_) of the phenotypic variation for αT in the POP-1, respectively. In POP-2 phenotypic variation of 52.75% (09BJF_2:3_), 12.44% (09HNF_2:4_) and 39.09% (10HBF_2:4_) for αT were observed. The significant effects indicated that the *qd5-1* QTL could be a qualitative gene and can be fine mapped by analyzing the recombinants among the segregating populations.

**Figure 3:**
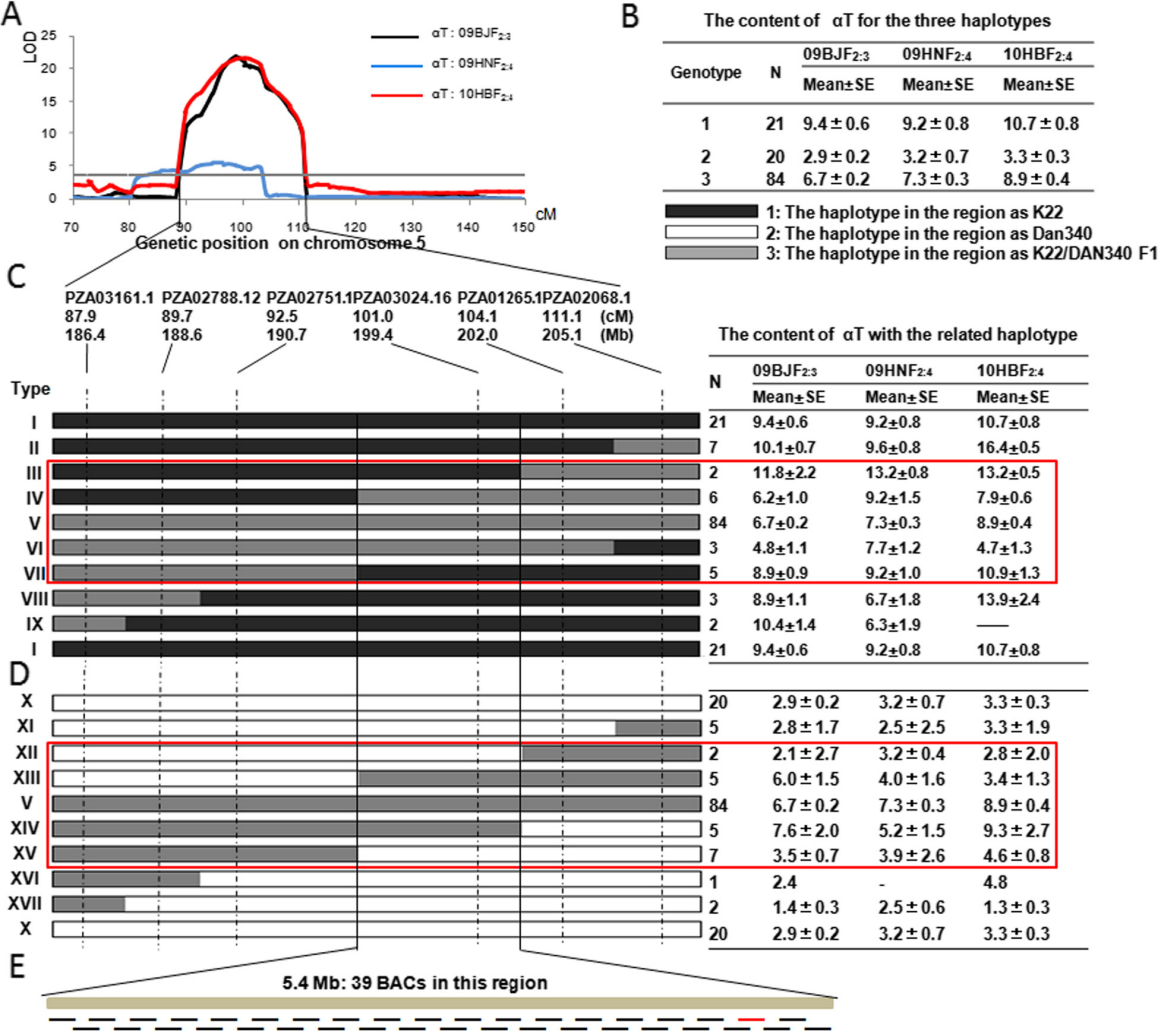
**Haplotype analyses and fine-mapping of *qd5-1 *in the early generation in POP-2 (A) Map position of *qd5-1 *in three environments.** (**B**) Summary of genotype and phenotypic effects in the genomic regions that contains *qd5-1*. (**C**, **D**) The detailed haplotype analyses between types 1 and 3 (**C**) or types 2 and 3 (**D**) as in Figure 3B. The bars indicate the missing data. (**E**) The distribution of BACs (black lines) and candidate genes (red line indicates *VTE4*) in the genomic regions after fine mapping. The black lines mean each BAC and the red line represent the BAC that the candidate gene *VTE4* located, all the BACs information is from web site ( http://www.maizesequence.org/ B73_version 5a.60)*,*.

The POP-2 was first selected to analyze recombination within the *qd5-1* region because of its wide phenotypic variation for αT. From the initial mapping, the PZA03161.1 and PZA02058.1 markers were located at either end of the region separately by 25.2cM genetic distance and 18.7 Mb (186.4-205.1 Mb) physical distances. There were four additional markers within this region (Figure 3A). In total, five recombinant combinations were identified, dividing the 180 RILs (after deleting some ambiguous RILs) into 17 haplotypes (Figure 3C and D). The 17 haplotypes could be divided into 3 obvious groups based on 09BJF_2:3_ data: group 1 for haplotypes IV, V, VI, XIII, and XIV (Figure 3C; 4.8 - 7.6 ug/g, αT), group 2 for haplotypes I, II, III, VII, VIII and IV (8.9 - 11.8 ug/g, αT) and group 3 for the rest haplotypes (− 1.4 - 2.9 ug/g, αT). Similar trends were also observed in the F_2:4_ populations from the other two locations (Figure 3C and D). Comparing the phenotypes of type 1 (haplotype I from the parent K22 I in Figure 3C), type 3 (haplotype V from the K22/Dan340 F1 in Figure 3C) and type 2 (haplotype X from the parent Dan340; Figure 3C) indicated that the parent K22 had the favorable allele for αT in the QTL region (Figure 3B). The phenotype of group 1 was similar to type 3, the group 2 was similar with type 1 and group 3 phenotype was similar with type 2. Based on this information, the QTL region was narrowed down between markers PZA02751.1 and PZA01265.1 to around 10.8 Mb region. There are 39 bacterial artificial chromosomes (BACs) identified within this target region and included an annotation for the *VTE4* tocopherol biosynthesis gene.

Using the same approach, another QTL-*qd1-1* in POP-2, which explained 30.81% of γT content, was analyzed. All the families were divided into 19 haplotypes and the haplotypes of I, II, III. IX, X, XI, and XII had the lowest γT content. Consistent with our expectation, the interval could narrowed from 21.2 cM (23.1 Mb, 218.1-241.2Mb) to ~6.5 cM (~5.2 Mb) in POP-2 map (Additional file 1: Figure S2).

## Discussion

### The genetic basis for tocopherol biosynthesis in maize grain

In the present study, 30 QTLs were detected in two F_2:3_ populations, with one to six QTLs for each trait. These QTLs can explain the phenotypic variation of each trait from 23.4% for α/γ in POP-1 to 66.5% for TT in POP-2. Two major QTLs (*qc5-1* and *qc5-2*) that affect all four target traits were identified in POP-1. Both *qc5-1* and *qc5-2* were located on chromosome 5 and explained a maximum phenotypic variation of 30% and 25% for αT, respectively (Table 3). In POP2, the major QTLs one (*qd1-1*) on chromosome 1 and the *qd5-1* QTL on chromosome 5 explained 51% (TT) and53% (αT) phenotypic variation respectively (Table 4). More than 80% of the QTLs detected in this study contributed in an additive manner. Hence, vitamin E biosynthesis in maize grain may be controlled by several major and a number of minor QTLs. This phenomenon is different with other agronomic traits, such as flowering time [36] and leaf architecture [37]. These traits are controlled by many minor QTLs and to data no major QTLs have been reported. Vitamin E is beneficial for human and animal health, but the absence of a selection index makes it difficult to breed for this trait, which is not as important compared as other economic traits such as yield. This type of trait may not be under strong selection pressure in the breeding and farming community, which is why major QTLs were easily identified. With the advent of such major additive QTLs the total tocopherols content of maize grains can be easily increased using marker assistant selection (MAS), which can be a simple guide for breeders to improve such traits. Furthermore, fine mapping of these major QTLs will explore the phenomenon in a comprehensive way because only a few candidate genes were detected in each QTL region, which distinguishes this pathway in maize from the extensively studied corresponding one in *Arabidopsis*. Hence, the study of tocopherol compounds in maize grains will help to increase the economic and nutritional value of maize crops which could account for more than half of the increased worldwide consumer demand for cereals [38].

When a QTL with a large effect is identified, determining the causal gene is a tedious and time-consuming task [39], although the involvement of a few major genes, facilitates the identification and fine mapping of the candidate gene. In addition, a single large-effect QTL often has multiple, closely linked QTLs with smaller, and sometimes opposite, effects on the phenotype [40,41]. The *qc5-1/qd5-1* QTL has a large effect on the overall phenotype [31,32]. Table 5 shows some QTLs that were identified in previous study [32]. A comparison of the results shows that *qd1-1* was only in POP-2, which is similar to the previous studies of chromosome 8 [32]. The populations used in this study are of different genetic background as compared with those in the previous studies, so the source may be different across the different genetic material. However, our analysis has resulted in better coverage of the maize genome by using a large number of markers to determine more possible QTLs playing any kind of major or minor role in this phenomenon.

**Table 5:** Comparison of QTLs detected in this study and in the study of Chander’s study

**QTL_name**	**Results described in this study**	**Results from Chander et al. **[32]		
	**Position**	**Trait**	**Position**	**Trait**
*qc1-1*	55.6-66.0Mb	αT, TT	51.5-69.7Mb	αT, γT, TT
*qc2-2*	4.9-9.4Mb	TT	2.6-5.5Mb	αT, γT, TT
*qc5-1/qd5-1*	8.2-34.6Mb	αT, γT, TT	12.0-33.0Mb	γT, TT, α/γ
*qc6-1*	9.6-81.8Mb	γT	77.7-95.0Mb	δT
*qc7-1*	103.4-128.4Mb	γT, TT	85.0-134.0Mb	δT

It is interesting to note that the two candidate genes *VTE4* and *HPPD-5* from the tocopherol biosynthesis pathway were both located within one corresponding QTL region in this study, it suggested that the genetic system controlling the biosynthesis of tocopherol may be more complex in maize as compared with that in *Arabidopsis*. Hence, the tocopherol biosynthesis pathway should be explored in more maize populations with different genetic backgrounds. Further studies will also help to identify the exact number of QTLs with minor or major roles, as a large population size and high number of markers are required to provide a solid basis for further improvement. Finally, positional cloning of major QTLs is extremely important to validate the results described herein and to improve the overall performance of maize.

### Regulation of tocopherol synthesis

Many biosynthetic genes control the rate of synthesis of tocopherol. Nine of the downstream genes have been thoroughly studied in *Arabidopsis*. *VTE1*, *VTE2*, *VTE3*, *VTE4*, *VTE5* and *HPPD* are involved in the formation of the end-product, whereas *GGPS*, *DXS*, *DXR* and *VTE5* are involved in synthesis of the tocopherol precursor (Figure 1). Maize homologous genes of the first six genes were identified through bioinformatics, with some genes having more than one copy in maize. The physical positions and abbreviated names of the enzyme and chromosome location of these candidate genes are given in B73 (Additional file 1: Table S3, http://www.maizesequence.org/, B73_version 5a.60).

Several QTLs co-localized with corresponding candidate genes, and just one QTL and candidate gene was found in more than one location (Tables 3 and 4). On chromosome 5, *VTE4* were co-located in both maps, whereas *HPPD-5* was found in only one population. The large QTL on chromosome 1 in POP-2 had no candidate gene, suggesting that the presence of additional unknown genes controlling tocopherol content have not been identified by comparative genomics.

### How to mine genes with large QTLs in the future

The rapid development of the high-throughput SNP genotyping technique enables the easy construction of simple high-density linkage maps. In the present study, the linkage map constructed by SNP markers was 1389.3 cM in POP-1 and 1567.5 cM in POP-2 with an average interval distance of 3.25 and 4.57 cM, respectively (average interval distance was 2.67 cM in the consensus map). Previously, linkage maps were constructed by SSR markers with an interval distance of 10–30 cM in maize and varying distance for different organism [42]. High-density maps more precisely localize major QTLs to smaller region.

Fine-mapping requires the construction of advanced backcrossing populations and high-density markers to narrow down the QTL region to one gene or even a single SNP [43]. Fine mapping and functional validation are usually more costly, laborious and time consuming. Recently, the rapid development of association mapping has enabled the identification of a single gene within a year of collecting phenotypic and genotypic data. It provides a new tool for analyzing quantitative traits. Li et al. [44] combined traditional fine mapping and association mapping to identify the functional gene *fatb* and validated the functional sequence variation using *in vivo* gene expression profiling and *in vitro* complementation studies. They developed markers based on the predicted gene sequences and increased marker density to narrow down the region of interest, and also performed an association analysis with these markers in 74 lines. The combined linkage and association mapping is therefore a beneficial tool for identifying novel genes for different qualitative and quantitative traits [44].

We performed fine mapping for the large *qd5-1* and *qd1-1* by haplotype analysis in an early generation and obtained good results without constructing a large backcross population. This method allowed the large QTL *qd5-1* region to be narrowed from 18.7 Mb to 5.4 Mb, assuming that the recombination occurred in the middle of the markers. There were 39 BACs in this region, with *VTE4* – *GRMZM2G035213* found in BAC209363 using bioinformatic analysis (Figure 3E, http://www.maizesequence.org/, B73_version 5a.60). Recently, *VTE4* was identified as the underlying gene of this QTL using genome-wide and candidate gene association analyses [45]. Two functional polymorphisms (InDel7 and InDel118) were significantly associated with αT. InDel7 segregates in the parents of the two populations. InDel118 segregates in K22 and Dan340 populations. Hence, CI7 has the best haplotype (7/118), DAN340 has the worst haplotype (0/0), and the haplotype of K22 (7/0) is intermediate, which is also consistent with the phenotype of the three parents and QTL effects in the two populations. The cloned gene *VTE4* provides an excellent sample of the high-density markers for QTL fine mapping and cloning.

The size of the *qd1-1*, QTL diminished from 23.1 Mb to 2.6 Mb, and there were 22 BACs in the 2.6 Mb region without any known candidate genes (Additional file 1: Figure S2E). Further research is needed to validate the functional site of these QTLs. There might be more genes controlling the tocopherol degradation pathway than the genes known to be involved in the synthesis pathway. Hence, much more in-depth work is needed for dissecting the metabolic pathway of tocopherols in maize grain including the synthesis and degradation pathways.

## Conclusion

This study identified different major QTLs in different populations compared with previous studies [32]. Thoroughly understanding the genetic architecture of tocopherol biosynthetic and degradation pathway is required to construct more populations with different genetic backgrounds. According to the recent methodology of combining linkage and association mapping [44], dissecting the tocopherol pathway can be performed in a short period of time with maximum validations, thus providing the scientific community with a base for MAS. MAS may be a useful and cost-effective tool for improving the nutritional value of the world’s leading cereal. In the preceding decades many major QTLs for different traits were applied in breeding programs by developing some functional markers [46,47]. Similarly, the three major QTLs in this study (*qd1-1*, *qc5-1*/*qd5-1* and *qc5-2*) can provide a guide for the development of molecular markers for breeding program or further detailed and deep research.

## Methods

### Genetic materials

An elite Chinese inbred line K22 was chosen to cross with two other elite lines, CI7 and Dan340, which have significantly different tocopherol contents [48]. Four hundred kernels of each F_2_ population were planted to develop the F_2:3_ population by self-pollinating at the Changping experiment field of China Agricultural University in Beijing (spring, 2009). Thirteen individuals were grown in a 3-meters row with 0.5-meter spacing within the row. Ears were harvested after 40–45 days of pollination, and 237 F_2:3_ families of POP-1 and 218 F_2:3_ families of POP-2 were obtained and phenotyped, diseased and contaminated ears were excluded from analysis. These F_2:3_ families were used for phenotyping and validation in offspring. Trials were conducted at two locations with two replications per location. At one location, the F_2:3_ families were planted in the Nanbin farm in Yacheng of Hainan province with 11 plants in each 3-meters row (winter, 2009). Another trial was carried out in Hubei Academy of Agricultural Sciences with the same field design as that in Beijing in 2009 (spring, 2010). Pooled pollen from the line was used to pollinate at least five plants so as to harvest good ears for phenotyping. Finally, 189 (POP-1) and 198 (POP-2)F_2:4_ families were measured in Hainan, and 213 (POP-1) and 177 (POP-2) F_2:4_ families were measured in Hubei because of asynchronized flowering and additional developmental problems.

### Reagents

The standards for γT, αT, δT and other chromatography-grade chemicals were purchased from Sigma (St Louis, MO, USA) and all other chemicals from Beijing Chemical Reagent Factory (Sinopharm Group Chemical Reagent Co., Ltd, Beijing, China).

### Measurement of tocopherols

At physiological maturity the ears were harvested and shelled manually, and a sample of 50 well performed was taken for phenotyping. These kernels were selected from the middle of each F_2:3_ or F_2:4_ ears, by bulk-pollinated in each family, respectively. All the kernels used for phenotypic analysis were dried for 60 hours at 45°C, kept in the dark at 4°C and ground into powder for tocopherol extraction and measurement. Tocopherols were extracted with the modified method as described in previous studies [32,48,49]. Three metabolites γT, αT and δT were measured separately. In addition to αT and γT, two derived traits, TT (the sum of γT, αT and δT) and α/γ (the ratio of α-/γ-tocopherol) were also calculated.

The tocopherol content was determined by high performance lipid chromatography (HPLC) as described [32,48,49]. External standard curves were constructed with eight serial dilutions and with repeats for each dilute (R^2^ ≥ 0.99). The three tocopherols (γT, αT and δT) were separated on a reverse-phase C30 column (YMC CT99S05-2546WT C30, 4.6nm × 25cm, 5μm; Waters) at 30°C at 1.8 ml/min for the mobile phase (v/v/v, 75:20:5; acetonitrile/methanol /dichloromethane) by scanning at 295 nm without a reference wave and were identified by the retention time of the standards. The peak times for δT, γT and αT were 4.56 min, 5.15 min and 6.07 min, respectively. All phenotypic data were generated on ChemStation software (Agilent Technologies).

### Phenotypic data analysis

The variance of traits and the correlation coefficients between traits were analyzed using the “PROC GLM” procedure of SAS 8.02 (SAS Institute 1999). The broad sense heritability was estimated as *h*^*2*^ =σ_g_^2^ / (σ_g_^2^ +σ_gy_^2^+σ_e_^2^/y) with SAS 8.02. Here, σ_g_^2^ is the genetic variance, σ_gy_^2^ is the interaction of genotype with year, σ_e_^2^ is the residual error, and y is the number of years [50]. All the traits were analyzed with the same method.

### Genotyping and linkage map construction

Genomic DNA was extracted from all 455 F_2_ single plants (237 lines of POP-1 and 218 lines of POP-2) and from their parental genotypes using the modified procedure of Murry and Thompson [51]. All the families and parents were genotyped using the GoldenGate assays (Illumina, San Diego, CA, USA) containing 1,536 SNPs [34]. The SNP genotyping was performed on an Illumina BeadStation 500G at Cornell University Life Sciences Core Laboratories Center using the protocol supported by Illumina Company [52]. The details of the SNP genotyping procedure and allele scoring have been described [34]. The data from polymorphic SNPs were used to construct a genetic linkage map using Mapmaker 3.0 for each population [53]. The threshold LOD score for the test of independence of marker pairs was set at 3.0, and the marker order with the highest LOD score was then selected. The Kosambi mapping function was used for calculating map distances. The two individual maps were then combined to form a consensus map using merge map [35].

### QTL analysis

For QTL detection the whole genome was scanned using composite interval mapping (CIM) with 2 cM scanning intervals between markers and a windows size of 10 cM. We used Model 6 in the Zmapqtl module of Wincartographer 2.5 [54]. The threshold LOD values for putative QTLs for tocopherol content and composition were estimated after 1,000 permutations at a significant level of p < 0.05 [55]. The number of marker cofactors for the background control was set by forward–backward stepwise regression with five controlling markers.

## Abbreviations

γT: γ-tocopherol; αT: α-tocopherol; α/γ: α-tocopherol/γ-tocopherol; TT: Total tocopherol; SSR: Simple sequence repeat; SNP: Single nucleotide polymorphism; QTL: Quantitative trait loci; POP-2: K22/Dan340 population; POP-1: K22/CI7 population; MAS: Marker assistant selection; LOD: Logarithm of odds; HPLC: High performance lipid chromatography; *h*^*2*^: The broad sense heritability; chr: Chromosome; 10HBF2:4: F_2:4_ populations in Hubei 2010; 09HNF2:4: F_2:4_ populations in Hainan 2009; 09BJF2:3: F_2:3_ populations in Beijing 2009.

## Competing interests

The authors declare no completing financial interests.

## Authors’ contributions

XST and ZDL prepared the materials; XST, ZDL LQ and CY performed the analyses of the genotype and phenotype; LZG and WWD participated to determine the phenotype; ST constructed the linkage maps; YJB, LJS and YXH designed the experiments and analyzed the chip raw data; XST, FA and YJB wrote the manuscript. All authors read and approved the final manuscript.

## Supplementary Material

Additional file 1 **Table S1.** Correlation coefficients of trait pairs for tocopherol related traits in two segregating populations in three environments. **Table S2.** Correlation coefficients of each trait among three locations. **Table S3.** List of candidate genes related to tocopherols in maize grains compared with that in *Arabidopsis.***Figure S1.** The entire linkage map of chromosome 1-chromosome 10. **Figure S2.** Haplotype analyses and fine mapping of *qd1-1* in the early generation of K22/Dan340 segregation population A: Map position of *qd1-1* in three environments, 2009 Beijing, 2009 Hainan and 2010 Hubei. B: Summary of genotype and phenotype in the genomic regions harboring *qd1-1*. C: The detailed haplotype analyses between type 1 and type 3 as Additional file 1: Figure S2B. The bar means the missing data. D: The detailed haplotype analyses between type 2 and type 3 as Additional file 1: Figure S2B. The bar means the missing data. The bar means the missing data. E: The distribution of BACs and candidate genes in the genomic regions after fine mapping. The black lines mean each BAC. All the BACs information is from web site ( http://www.maizesequence.org/i B73 RefGen_v2). Click here for file

## References

[B1] Bramley PM Elmadfa I Kafatos A Kelly FJ Manios Y Roxborough HE Schuch W Sheehy PJA Wagner K-H Critical reviews produced within the EU Concerted Action 'Nutritional enhancement of plant-based food in European trade' (Neodiet) – Vitamin E Sci Food Agri 2000 80 913 938

[B2] Rocheford TR Wong JC Egesel CO Lambert RJ Enhancement of Vitamin E Levels in Corn J Amer Coll Nutr 2002 21 191 198 10.1080/07315724.2002.10719265 12071304

[B3] Moss GP Nomenclature of tocopherols and related compounds. Recommendations 1981 Eur J Biochem 1981 123 473 475 7075595

[B4] DellaPenna D A decade of progress in understanding vitamin E synthesis in plants J Plant Physiol 2005 162 729 737 16008096 10.1016/j.jplph.2005.04.004

[B5] Traber MG Atkinson J Vitamin E, antioxidant and nothing more Free Rad Biol & Med 2007 43 1 4 15 17561088 10.1016/j.freeradbiomed.2007.03.024 PMC2040110

[B6] Herrera E Barbas C Vitamin E: action, metabolism and perspectives J Physio and Biochem 2001 57 2 43 56 11579997

[B7] Traber MG Sies H Vitamin in humans: demand and delivery Annual Review Nutr 1996 16 321 347 10.1146/annurev.nu.16.070196.001541 8839930

[B8] Institute of Medicine Food and Nutrition Board: Dietary reference intakes: Applications in dietary assessment 2000 National Academy Press, Washington, DC 289

[B9] Sokol RJ Vitamin E deficiency and neurologic disease Annual Review Nutr 1988 8 351 373 10.1146/annurev.nu.08.070188.002031 3060170

[B10] Aicardi J Diseases of the nervous system in childhood Mac Keith Press 1992 108 202

[B11] Eggermont E Recent advances in vitamin E metabolism and deficiency Eur J Pediatr 2006 165 429 434 16491383 10.1007/s00431-006-0084-5

[B12] Muller DPR Vitamin E and neurological function Mol Nutr Food Res 2010 54 710 718 20183831 10.1002/mnfr.200900460

[B13] Norris SR Barrette TR DellaPenna D Genetic dissection of carotenoid synthesis in Arabidopsis defines plastoquinone as an essential component of phytoene desaturation Plant Cell 1995 7 2139 2149 8718624 10.1105/tpc.7.12.2139 PMC161068

[B14] Norris SR Shen XH DellaPenna D Complementation of the Arabidopsis pds1 mutation with the gene encoding phydroxyphenylpyruvate dioxygenase Plant Physiol 1998 117 1317 1323 9701587 10.1104/pp.117.4.1317 PMC34895

[B15] Porfirova S Bergmüller E Tropf S Lemke R Dörmann P Isolation of an Arabidopsis mutant lacking vitamin E and identification of a cyclase essential for all tocopherol biosynthesis Proc Natl Acad Sci. USA 2002 99 12495 12500 12213958 10.1073/pnas.182330899 PMC129473

[B16] Sattler SE Cahoon EB Coughlan SJ DellaPenna D Characterization of tocopherol cyclases from higher plants and cyanobacteria. Evolutionary implications for tocopherol synthesis and function Plant Physiol 2003 132 2184 2195 12913173 10.1104/pp.103.024257 PMC181302

[B17] Collakova E DellaPenna D Isolation and functional analysis of homogentisate phytyltransferase from Synechocystis sp. PCC 6803 and Arabidopsis Plant Physiol 2001 127 1113 1124 11706191 PMC129280

[B18] Schledz M Seidler A Beyer P Neuhaus G A novel phytyltransferase from Synechocystis sp. PCC 6803 involved in tocopherol biosynthesis FEBS Lett 2001 499 15 20 11418103 10.1016/s0014-5793(01)02508-x

[B19] Savidge B Weiss JD Wong YHH Lassner MW Mitsky TA Shewmaker CK Post-Beittenmiller D Valentin HE Isolation and characterization of homogentisate phytyltransferase genes from Synechocystis sp. PCC 6803 and Arabidopsis Plant Physiol 2002 129 321 332 12011362 10.1104/pp.010747 PMC155895

[B20] Sattler SE Gilliland LU Magallanes-Lundback M Pollard M DellaPenna D Vitamin E is essential for seed longevity and for preventing lipid peroxidation during germination Plant Cell 2004 16 1419 1432 15155886 10.1105/tpc.021360 PMC490036

[B21] Cheng Z Sattler S Maeda H Sakuragi Y Bryant DA DellaPenna D Highly divergent methyltransferases catalyze a conserved reaction in tocopherol and plastoquinone synthesis in cyanobacteria and photosynthetic eukaryotes Plant Cell 2003 15 2343 2356 14508009 10.1105/tpc.013656 PMC197300

[B22] Motohashi R Ito T Kobayashi M Taji T Nagata N Asami T Yoshida S Yamaguchi-Shinozaki K Shinozaki K Functional analysis of the 37 kDa inner envelope membrane polypeptide in chloroplast biogenesis using a Ds-tagged Arabidopsis pale-green mutant Plant J 2003 34 719 731 12787252 10.1046/j.1365-313x.2003.01763.x

[B23] Shintani D DellaPenna D Elevating the vitamin E content of plants through metabolic engineering Science 1998 282 2098 2100 9851934 10.1126/science.282.5396.2098

[B24] Ischebeck T Zbierzak AM Kanwischer M Dörmann P A salvage pathway for phytol metabolism in Arabidopsis J Biol Chem 2006 281 2470 2477 16306049 10.1074/jbc.M509222200

[B25] Valentin HE Lincoln K Moshiri F Jensen PK Qi Q Venkatesh TV Karunanandaa B Baszis R Norris SR Savidge B Gruys KJ Last RL The Arabidopsis vitamin E pathway gene5-1 Mutant Reveals a Critical Role for Phytol Kinase in Seed Tocopherol Biosynthesis Plant Cell 2006 18 212 224 16361393 10.1105/tpc.105.037077 PMC1323494

[B26] Zou LP Rice γ-tocopherol methyltransferase gene cloning and analysis of full-length cDNA Hubei Agri Sci 2008 47 11 1221 1224

[B27] Zou LP Cloning and sequence analysis γ-tocopherol methyltransferase (γ-TMT) gene in tomato J Anhui Agri Sci 2008 36 2 437 439

[B28] Shintani DK Cheng ZG DellaPenna D The role of 2-methyl-6-phytylbenzoquinone methyltransferase determining tocopherol composition in Synechocystis sp. PCC6803 FEBS Lett 2002 511 1 5 11821038 10.1016/s0014-5793(01)03223-9

[B29] Marwede V Gui MK Becker HC Ecke W Mapping of QTL controlling tocopherol content in winter oilseed rape Plant Breeding 2005 124 20 26

[B30] Vera-Ruiz EM Velasco L Leon AJ Fernández-Martínez JM Pérez-Vich B Genetic mapping of the Tph1 gene controlling beta-tocopherol accumulation in sunflower seeds Molecular Breeding 2006 17 291 296

[B31] Wong JC Lambert RJ Tadmor Y Rocheford TR QTL associated with accumulation of tocopherols in maize Crop Sci 2003 3 2257 2266

[B32] Chander S Guo YQ Yang XH Yan JB Zhang YR Song TM Li JS Genetic dissection of tocopherol content and composition in maize grain using quantitative trait loci analysis and the candidate gene approach Mol Breeding 2008 22 353 365

[B33] Yan JB Shah T Warburton ML Buckler ES McMullen MD Crouch JH Genetic characterization and linkage disequilibrium estimation of a global maize collection using SNP markers PLoS One 2009 4 12 e8451 20041112 10.1371/journal.pone.0008451 PMC2795174

[B34] Yan JB Yang XH Shah T Sa’nchez-Villeda H Li JS Warburton ML Zhou Y Crouch JH Xu YB High-throughput SNP genotyping with the GoldenGate assay in maize Mol Breeding 2010 25 441 451

[B35] Wu YH Bhat PR Close TJ Lonardi S On the Accurate Construction of Consensus Genetic Maps CSB 2008 - Computational Systems Bioinformatics Conference Stanford, CA http://alumni.cs.ucr.edu/~yonghui/mgmap.html 19642288

[B36] Buckler ES Holland JB Bradbury PJ Acharya CB Brown PJ Browne C Ersoz E Flint-Garcia S Garcia A Glaubitz JC Goodman MM Harjes C Guill K Kroon DE Larsson S Lepak NK Li HH Mitchell SE Pressoir G Peiffer JA Rosas MO Rocheford TR Romay MC Romero S Salvo S Villeda HS Silva HS Sun Q Tian F Upadyayula N Ware D Yates H Yu JM Zhang ZW Kresovich S Michael D McMullen MD The genetic architecture of maize flowering time Science 2009 325 5941 714 718 19661422 10.1126/science.1174276

[B37] Tian F Bradbury PJ Brown PJ Hung H Sun Q Flint-Garcia S Rocheford TR McMullen MD Holland JB Buckler ES Genome-wide association study of leaf architecture in the maize nested association mapping population Nat Genet 2011 43 159 162 21217756 10.1038/ng.746

[B38] Yan JB Warburton ML Crouch J Association mapping for enhancing maize genetic improvement Crop Sci 2011 51 433 449

[B39] Ingvarsson PK Street NR Association genetics of complex traits in plant New Phytol 2011 189 909 922 21182529 10.1111/j.1469-8137.2010.03593.x

[B40] Doerge RW Mapping and analysis of quantitative trait loci in experimental populations Nat Rev Genets 2002 3 43 52 10.1038/nrg703 11823790

[B41] Mackay TFC Stone EA Ayroles JF The genetics of quantitative traits: challenges and prospects Nat Rev Genet 2009 10 565 577 19584810 10.1038/nrg2612

[B42] Salvi S Tuberosa R To clone or not to clone plant QTLs, present and future challenges Trends Plant Sci 2005 10 297 304 15949764 10.1016/j.tplants.2005.04.008

[B43] Remington DL Thornsberry JM Matsuoka Y Wilson LM Sherry R Whitt SR Doebley J Kresovich S Goodman MM Buckler ES Structure of linkage disequilibrium and phenotypic associations in the maize genome Proc Natl Acad Sci 2001 98 11479 11484 11562485 10.1073/pnas.201394398 PMC58755

[B44] Li L Li L Li Q Yang XH Zheng DB Warburton ML Chai YC Zhang P Guo YQ Yan JB Li JS An 11-bp Insertion in Zea mays fatb reduces the palmitic acid content of fatty acids in maize grain PLoS One 2011 6 9 e24699 21931818 10.1371/journal.pone.0024699 PMC3172307

[B45] Li Q Yang XH Xu ST Cai Y Zhang DL Han YJ Li L Zhang ZX Gao SB Li JS Yan JB Genome-wide association studies identified three independent polymorphisms associated with α-tocopherol content in maize kernels PLoS One 2012 7 5 e36807 22615816 10.1371/journal.pone.0036807 PMC3352922

[B46] Cao SL Development of application with marker assisted-selection in maize breeding Crops 2008 6 107 109

[B47] Yang H Yang JP Rong TZ Wang FG Tan J Qiu ZG Developing the maize lines based on selections of phi116 and umc1044 markers which are resistant to sheath blight Mol Plant Breeding 2007 5 3 347 352

[B48] Zhou Y Fu ZY Li Q Xu ST Chander S Yang XH Li JS Yan JB Comparative analysis of carotenoid and tocopherol compositions in high-oil and normal maize (Zea mays L.) inbreds Acta Agronomica Sinica 2009 35 2073 2084

[B49] Kurilich AC Juvik JA Quantification of carotenoid and tocopherol antioxidants in Zea mays J Agric Food Chem 1999 47 1948 1955 10552476 10.1021/jf981029d

[B50] Knapp SJ Stroup WW Ross WM Exact confidence intervals for heritability on a progeny mean basis Crop Science 1985 25 192 194

[B51] Murry MG Thompson WF Rapid isolation of high molecular weight plant DNA Nucleic Acids Res 1980 8 4321 4325 7433111 10.1093/nar/8.19.4321 PMC324241

[B52] Fan JB Gunderson KL Bibikova M Yeakley JM Chen J Wickham Garcia E Lebruska LL Laurent M Shen R Barker D Illumina universal bead arrays Methods Enzymol 2006 410 57 73 16938546 10.1016/S0076-6879(06)10003-8

[B53] Lincoln SE Daly MJ Lander ES Construction genetic maps with MAPMAKER/EXP 3.0. Whitehead Institute Technical Report, White-head Institute, Cambridge, Massachusetts 1993 3

[B54] Zeng ZB Precision mapping of quantitative trait loci Genetics 1994 136 1457 1468 8013918 10.1093/genetics/136.4.1457 PMC1205924

[B55] Churchill GA Doerge RW Empirical threshold values for quantitative trait mapping Genetics 1994 138 963 971 7851788 10.1093/genetics/138.3.963 PMC1206241

