# Supplementary material for: Dissecting tocopherols content in maize (Zea mays L.), using two segregating populations and high-density single nucleotide polymorphism markers
Source: BMC Plant Biol. 2012 Nov 2;12:201. doi: 10.1186/1471-2229-12-201 (PMC3502391; doi:10.1186/1471-2229-12-201)
Supplement: Additional file 1 — Table S1. Correlation coefficients of trait pairs for tocopherol related traits in two segregating populations in three environments. Table S2. Correlation coefficients of each trait among three locations. Table S3. List of candidate genes related to tocopherols in maize grains compared with that in Arabidopsis.Figure S1. The entire linkage map of chromosome 1-chromosome 10. Figure S2. Haplotype analyses and fine mapping of qd1-1 in the early generation of K22/Dan340 segregation population A: Map position of qd1-1 in three environments, 2009 Beijing, 2009 Hainan and 2010 Hubei. B: Summary of genotype and phenotype in the genomic regions harboring qd1-1. C: The detailed haplotype analyses between type 1 and type 3 as Additional file 1: Figure S2B. The bar means the missing data. D: The detailed haplotype analyses between type 2 and type 3 as Additional file 1: Figure S2B. The bar means the missing data. The bar means the missing data. E: The distribution of BACs and candidate genes in the genomic regions after fine mapping. The black lines mean each BAC. All the BACs information is from web site ( http://www.maizesequence.org/i B73 RefGen_v2). [file 1471-2229-12-201-S1.rtf]

Supplementary Tables and Figures

Supplementary Table 1 Correlation coefficients of trait pairs for tocopherol related traits in two segregating populations in three environments

Trait	Population	K22/CI7	K22/Dan340	
		ãT	áT	TT	á/ã	ãT	áT	TT	á/ã	
ãT	09BJF2:3	1	¡¡	¡¡	¡¡	1	¡¡	¡¡	¡¡	
¡¡	09HNF2:4	1	¡¡	¡¡	¡¡	1	¡¡	¡¡	¡¡	
¡¡	10HBF2:4	1	¡¡	¡¡	¡¡	1	¡¡	¡¡	¡¡	
áT	09BJF2:3	0.241**	1	¡¡	¡¡	-0.172*	1	¡¡	¡¡	
¡¡	09HNF2:4	0.248**	1	¡¡	¡¡	-0.268**	1	¡¡	¡¡	
¡¡	10HBF2:4	0.474**	1	¡¡	¡¡	-0.069	1	¡¡	¡¡	
TT	09BJF2:3	0.919**	0.603**	1	¡¡	0.961**	0.104	1	¡¡	
¡¡	09HNF2:4	0.919**	0.592**	1	¡¡	0.960**	0.012	1	¡¡	
¡¡	10HBF2:4	0.815**	0.896**	1	¡¡	0.875**	0.421**	1	¡¡	
á/ã	09BJF2:3	-0.678**	0.315**	-0.431**	1	-0.675**	0.510**	-0.542**	1	
¡¡	09HNF2:4	-0.677**	0.121	-0.503**	1	-0.597**	0.424**	-0.496**	1	
¡¡	10HBF2:4	-0.718**	0.037	-0.338**	1	-0.633**	0.435**	-0.370**	1	
** Significant at 0.01 probability, * Significant at 0.05 probability; 


Supplementary Table 2 Correlation coefficients of each trait among three locations
	
Populations	Generations	ãT	áT	TT	á/ã	
K22/CI7	09BJF2:3:09HNF2:4   	0.616**	0.498**	0.563**	0.548**	
¡¡	09BJF2:3:10HBF2:4 	0.507**	0.609**	0.559**	0.568**	
¡¡	09HNF2:4:10HBF2:4 	0.531**	0.494**	0.507**	0.482**	
K22/Dan340	09BJF2:3:09HNF2:4 	0.590**	0.348**	0.549**	0.543**	
¡¡	09BJF2:3:10HBF2:4 	0.775**	0.421**	0.698**	0.614**	
¡¡	09HNF2:4:10HBF2:4	0.496**	0.261**	0.471**	0.450**	
** Significant at 0.01 probability, 	


Supplementary Table 3 List of candidate genes related to tocopherols in maize grains compared with that in Arabidopsis	
¡¡	
Enzyme	Genea	Accessionsb	Chr	Positionc(Mb)	Arabidopsis ortholog(s)d	
4-hydroxyphenylpyruvate dioxygenase 1	HPPD-5	GRMZM2G088396	5	83.9	AT1G06570 (PDS1)[14]	
	HPPD-7	GRMZM2G374213	7	28.8		
tocopherol cyclase	VTE1	GRMZM2G009785	5	133.5	AT4G32770[15]	
homogentisate phytyltransferase	VTE2-2	GRMZM2G410644	2	199.9	AT2G18950[17]; AT3G11950[19]	
	VTE2-9	GRMZM2G048472	9	107.4		
	VTE2-9-2	GRMZM2G173358	9	92.5		
MPBQ/MSBQ methyltransferase	VTE3	GRMZM2G082998	1	174.0	AT3G63410[21]	
tocopherol O-methyltransferase;	VTE4	GRMZM2G035213	5	200.4	AT1G64970[23]	
gamma-tocopherol methyltransferase						
phytol kinase-related protein	VTE5-2	GRMZM2G104538	2	2.5	AT5G04490[25]	
	VTE5-4	GRMZM2G111632	4	183.4		
	VTE5-8	GRMZM2G046293	8	169.7		
 a abbreviation for the related gene in this paper; b accession number according to NCBI(http://blast.ncbi.nlm.nih.gov/); 
c physical positions for each BAC refer to maizesequence(http://www.maizesequence.org/, B73 RefGen_v2); 
d initial gene ID in Arabidopsis from TAIR(http://www.arabidopsis.org/); the superscripts of the gene ID mean the origin of the related gene. 


Supplementary Figure 1 The entire linkage map of chromosome 1-chromosome 10


Supplementary Figure 2 Haplotype analyses and fine mapping of qd1-1 in the early generation of K22/Dan340 segregation population A: Map position of qd1-1 in three environments, 2009 Beijing, 2009 Hainan and 2010 Hubei. B: Summary of genotype and phenotype in the genomic regions harboring qd1-1. C: The detailed haplotype analyses between type 1 and type 3 as Supplementary Figure 2B. The bar means the missing data. D: The detailed haplotype analyses between type 2 and type 3 as Supplementary Figure 2B. The bar means the missing data. The bar means the missing data. E: The distribution of BACs and candidate genes in the genomic regions after fine mapping. The black lines mean each BAC. All the BACs information is from web site (http://www.maizesequence.org/i B73 RefGen_v2).
